# Lower trunk kinematics and muscle activity during different types of tennis serves

**DOI:** 10.1186/1758-2555-1-24

**Published:** 2009-10-13

**Authors:** John W Chow, Soo-An Park, Mark D Tillman

**Affiliations:** 1 Center for Neuroscience and Neurological Recovery, Methodist Rehabilitation Center, Jackson, Mississippi, USA; 2 Department of Orthopedic Surgery, Asan Medical Center, University of Ulsan, Seoul, South Korea; 3 Department of Applied Physiology and Kinesiology, University of Florida, Gainesville, Florida, USA

## Abstract

**Background:**

To better understand the underlying mechanisms involved in trunk motion during a tennis serve, this study aimed to examine the (1) relative motion of the middle and lower trunk and (2) lower trunk muscle activity during three different types of tennis serves - flat, topspin, and slice.

**Methods:**

Tennis serves performed by 11 advanced (AV) and 8 advanced intermediate (AI) male tennis players were videorecorded with markers placed on the back of the subject used to estimate the anatomical joint (AJ) angles between the middle and lower trunk for four *trunk motions *(extension, left lateral flexion, and left and right twisting). Surface electromyographic (EMG) techniques were used to monitor the left and right rectus abdominis (LRA and RRA), external oblique (LEO and REO), internal oblique (LIO and RIO), and erector spinae (LES and RES). The maximal AJ angles for different trunk motions during a serve and the average EMG levels for different muscles during different phases (ascending and descending windup, acceleration, and follow-through) of a tennis serve were evaluated.

**Results:**

The repeated measures Skill × Serve Type × Trunk Motion ANOVA for maximal AJ angle indicated no significant main effects for serve type or skill level. However, the AV group had significantly smaller extension (p = 0.018) and greater left lateral flexion (p = 0.038) angles than the AI group. The repeated measures Skill × Serve Type × Phase MANOVA revealed significant phase main effects in all muscles (p < 0.001) and the average EMG of the AV group for LRA was significantly higher than that of the AI group (p = 0.008). All muscles showed their highest EMG values during the acceleration phase. LRA and LEO muscles also exhibited high activations during the descending windup phase, and RES muscle was very active during the follow-through phase.

**Conclusion:**

Subjects in the AI group may be more susceptible to back injury than the AV group because of the significantly greater trunk hyperextension, and relatively large lumbar spinal loads are expected during the acceleration phase because of the hyperextension posture and profound front-back and bilateral co-activations in lower trunk muscles.

## Introduction

Low back injuries are common among competitive tennis players [1-7]. General agreement exists that mechanical stress to the spine is related to the development of degenerative disc disease in the lumbar region [8]. Among different tennis strokes, the serve may place more stress on the lumbar spine than the other strokes because repetitive trunk hyperextension is generally thought to be the predisposing mechanism of spondylolysis [8-10]. Tennis players may be at an increased risk of lumbar disc pathology from rotational and hyperextension shearing effects [2]. The three types of serves that are widely used in tennis are the flat (minimum spin), topspin, and slice (sidespin) serves. In general, the flat serve is associated with a more forceful action and produces the fastest ball speed among the three types of serves. The spin involved in the topspin and slice serves permit the server to hit with greater accuracy. The racquet movement pattern and ball contact location relative to the body are different among these serves [11].

Because the spine is a complicated structure composed of many segments, joints, discs and various supporting muscles to protect the spinal cord and support the trunk mobility, it is very difficult to pinpoint the anatomic structures that cause low back pain. Identifying the biomechanical pathophysiologic factors associated with lumbar spinal loads may help to explain and prevent low back pathology. In national and world class tennis players who had structural disorders in their lumbar spines, Saal [12] reported a 3-to-1 ratio of disc to postelement syndromes. In addition, for the young tennis players, posterior element pain spondylolysis with or without spondylolisthesis compose the injury subset most frequently. Alyas et al. [13] found pars injuries and facet joint arthroses, predominately in the lower lumbar spine, to be relatively common in elite adolescent tennis players.

Motions of the trunk during occupational tasks have been identified as potential risk factors for developing low back disorders (LBD) in manual workers [14]. High values of combined trunk velocities (e.g., simultaneous lateral flexion and twisting velocities) were found to occur more often in high LBD risk jobs than in low LBD risk jobs. Dynamic strength of the trunk and structural loading are considered the two major contributing factors to the relationship between trunk dynamics and LBD. Structural loading factors include biomechanical factors that contribute to loading on the spinal structures such as intra-abdominal pressure, muscle activity, and the imposed trunk moment, and the actual loads on the structures of the spine.

Loading on the spinal structures can be very high during a tennis serve. This is especially true when the racquet moves behind the body and the vertebral column is laterally flexed and hyperextended. Acceleration of the racquet before ball impact is accompanied by a rapid reversal of the rotation of the lumbar spine - from hyperextension to flexion and right twist to left twist for a right-hander. This cork-screwing motion transfers the force of its torque to the spinal segments [15].

Body segmental and racquet kinematics of the tennis serve have been investigated extensively [16-18]. However, lumbar spine kinematics during the tennis serve have not been reported. Because low back injury is one of the most prevalent musculoskeletal diseases in tennis, a better understanding of the underlying mechanisms involved in trunk motion during a tennis serve is needed.

Information that identifies the muscles involved in stroke production is important for coaches and physical trainers [19]. Activity of the trunk muscles can be used to speculate on the stress on the lumbar intervertebral joints during dynamic tasks [20-23] and it has shown that the force generation and muscle recruitment activities associated with twisting change significantly as a function of the torso posture [24]. Prior EMG analyses of the tennis serve have focused on the muscles in the hitting arm, shoulder region, and lower extremity [25-31]. Very limited data on the activity of the lower trunk muscles during the tennis serve are available. Anderson [32] reported that both the left and right external obliques were very active (greater than 50% of the muscle's peak level of activity) during the force production phase of the tennis serve. In a preliminary study, Chow et al. [33] examined the muscle activation patterns of eight lower trunk muscles during flat, topspin, and slice serves in five male highly skilled tennis players. They found no major differences in muscle activation pattern across different serve types, and bilateral differences in muscle activation were more pronounced in rectus abdominis and external oblique than in internal oblique and lumbar erector spinae muscles. An appreciable amount of abdominal/low back and bilateral co-activation was observed during certain phases of the serve.

The purpose of this study was to evaluate the middle and lower trunk kinematics and selected lower trunk muscle activity during three different types of tennis serves (flat, topspin, and slice) in skilled male players. Because players struck the ball at a much more backward and to the left (for a right-hander) location on their second as compared to their first serve [11], players may need to arch backward and laterally flex more when executing a topspin serve. Therefore, it was hypothesized that greater spinal range of motion and lower trunk muscle activity would be found in the topspin serve when compared to the other serve types.

## Methods

### Subjects

Eleven advanced (AV) (United States Tennis Association National Tennis Rating Program (NTRP) 5.5, age 25.3 ± 4.1 years, height 180.3 ± 5.2 cm, mass 80 ± 8 kg) and eight advanced intermediate (AI) (4.5 - 5.0, 23.4 ± 6.5 years, 180.0 ± 9.5 cm, 78 ± 7 kg) male tennis players served as the subjects. NTRP ratings range from 1.0 (beginner) to 7.0 (world class professional). All subjects were right-handed and were in good physical condition and free of injury at the time of participation. The NTRP ratings were self-reported ratings. All subjects signed informed consent documents before their participation.

### Experimental Setup

**Figure 1:**
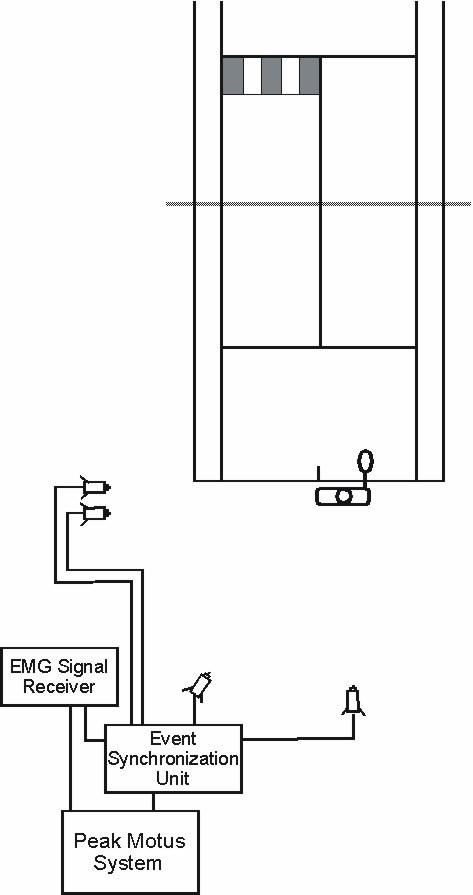
**Overhead view of the experimental setup**.

Four gen-locked video cameras (60 Hz) were stationed behind and to the left of the baseline of an indoor tennis court (Figure 1). One of the cameras was used to capture the whole body and racquet movements. The other three cameras captured the locations of reflective markers located on the back of the subject. A Peak event synchronization unit was used to synchronize the video and electromyographic recordings (Peak Performance Technologies, Inc., Inglewood, CO). For the purpose of spatial reference, a Peak calibration frame (1.5 m × 1.4 m × 1.3 m object space, 16 control points) was videotaped at the beginning of each data collection session. The frame was positioned at the baseline where the trunk of the subject was located during testing.

### Data Collection

Each collection session started with EMG trials followed by kinematics trials. Separate trials for EMG and kinematic data were needed because EMG electrodes interfered with reflective marker placement on the lower back.

#### EMG Trials

After jogging on a treadmill for five minutes as a warm-up, surface electrodes were attached to selected muscles of the lower trunk including the left and right rectus abdominis (3 cm lateral to the umbilicus), external obliques (approximately 15 cm lateral to the umbilicus), internal oblique (below the external oblique electrodes and just superior to the inguinal ligament), and lumbar erector spinae (3 cm lateral to L3 spinous process) [22]. The skin surfaces where the electrodes were located were cleansed with alcohol and shaved when necessary. Electrodes were placed over the bellies of each muscle parallel to the muscle's line of action with a center-to-center distance of 2.5 cm. Using a MESPEC 4000 telemetry system (Mega Electronics Ltd., Kuopio, Finland), the EMG signals were preamplified with a gain of 500 and band pass filtered at 8-1500 Hz (CMRR > 130 dB) close to the electrodes and telemetrically transmitted to a central receiver (gain = 1, Butterworth filter, 8-500 Hz band pass). The amplified EMG signals were sampled at 900 Hz (12-bit analog-to-digital conversion) using the Peak Motus system.

To obtain maximum EMG levels, two maximal isometric contractions were performed before the experimental trials - the bent-knee sit-up with the trunk inclined at approximately 30° to the horizontal and the trunk hyperextension performed in the prone position on a treatment table. In both maximal contractions, the feet were constrained and the resistance was applied manually at the shoulders. One trial was performed for each maximal contraction and each trial lasted for about 5 s.

After the isometric trials, the EMG transmitter was secured to the left hip of the subject using elastic bands. The subject was then asked to perform three different types of serves - flat, topspin, and slice. In all trials, the subject served with efforts comparable to his first serves during competition. They were asked to target their serves at the corner near the center line (flat and topspin) and sideline (slice). Seven trials were performed for each serve type and the serve type was presented in a random order. At the end of each trial, the subject was asked to rate his own performance based on the pace of the ball and landing location using a 5-point scale (5 = excellent, 0 = poor). For each trial, the EMG signals were collected for 5 s.

#### Kinematic Trials

**Figure 2:**
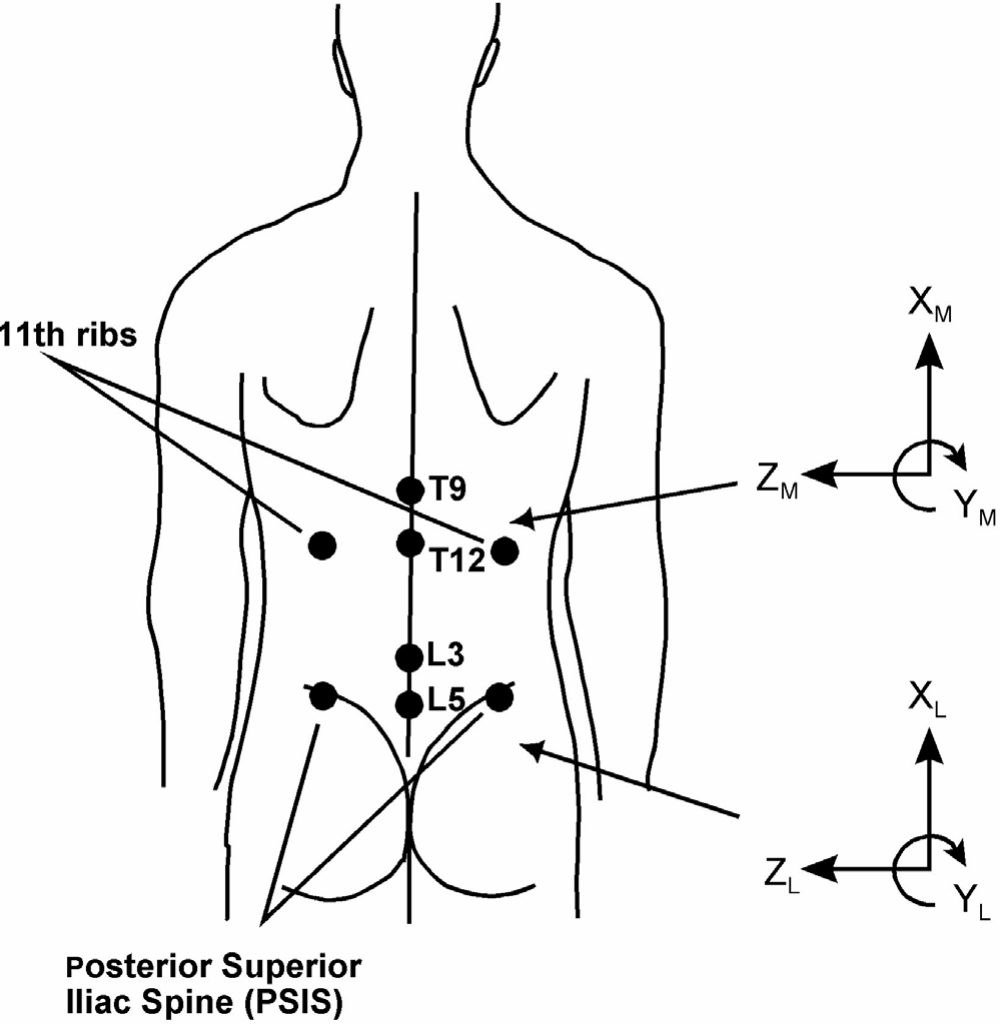
**Reflective marker locations and coordinate systems for the middle and lower trunk**.

After the EMG trials, electrodes were removed and eight reflective markers (each 1 cm in diameter) were placed on the back of the subject. The marker locations were right and left tips of the 11th rib, T9 and T12 spinous processes, right and left posterior superior iliac spines (PSISs), and L3 and L5 spinous processes (Figure 2). The location of the spine level was estimated using the techniques proposed by Tully and Stillman [34] and Schache et al. [35]. These markers were used to estimate the 3-dimensional (3D) orientations of the spinal regions directly above and below the lumbar spinal segments. These two regions were considered the middle and lower trunk for the purpose of this study. As a result, the change in relative motion between the middle and lower trunk was treated as motion in the lumbar spinal segments.

To establish the neutral orientation for the markers attached to each subject, the subject was asked to adopt a self-selected comfortable standing posture with arms folded in front of the torso while marker locations were recorded. The subject was then asked to perform seven trials for each type of serve and the order for the type of serve was assigned randomly. Again, the subjects served with efforts comparable to their first serves during competition and rated their own performance at the end of each trial.

### Data Reduction

**Figure 3:**
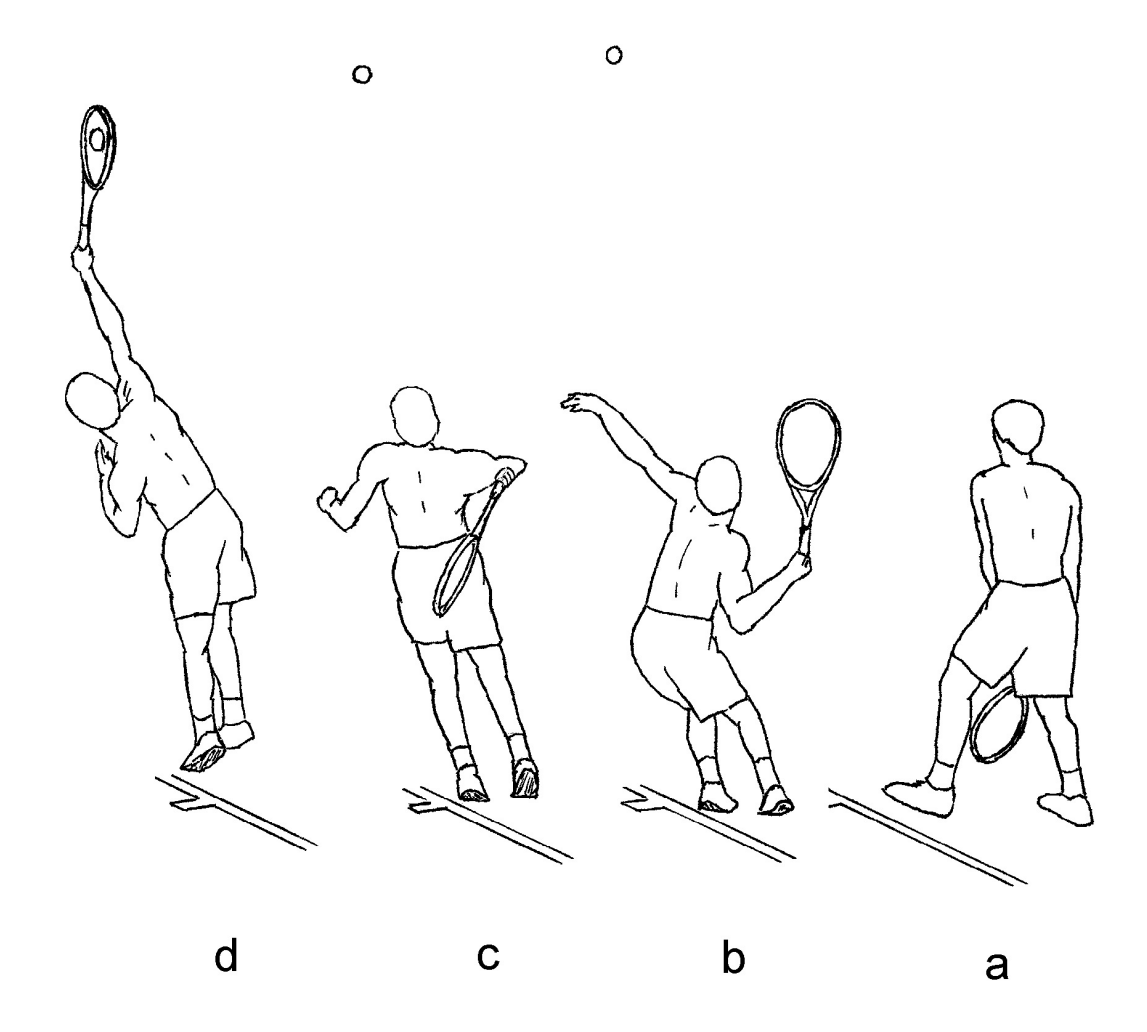
**Critical instants of a serve** -- (a) beginning of the windup, (b) end of the ascending windup, (c) end of the windup, and (d) ball impact. Adapted from Chow et al. [33].

For each subject, the two highest rated EMG and kinematics trials for each type of serve were analyzed. For each trial being analyzed, four critical instants were identified from the video recordings of whole body and racquet movements: (a) beginning of the windup, the instant when the racquet passed in front of the legs, (b) end of the ascending windup, the instant when the racquet reached the highest position during the windup, (c) end of the windup, the instant when the racquet reached the lowest position behind the trunk, and (d) ball impact. For the purpose of this study, the serve was divided into four phases (Figure 3):

• Ascending windup [instants (a) to (b)]

• Descending windup [instants (b) to (c)]

• Acceleration [instants (c) to (d)]

• Follow-through [0.1 s duration after instant (d)]

These phases are of interest because of the distinct movement and functional characteristics of the body and racquet during these phases.

#### EMG Data

The raw EMG signals were filtered using a recursive digital filter (Matlab Elliptic filter, 10-450 Hz band pass) and full-wave rectified. The maximum isometric trial data were smoothed using a moving average of 2 s and the largest average EMG value recorded for each muscle was considered the maximum EMG level. The experimental trial data were smoothed using a moving average of 50 ms before normalizing to the respective maximum EMG levels. An average normalized EMG value was computed for each muscle in each phase for each trial analyzed.

#### Kinematic Data

For each standing or serving trial analyzed, coordinate data were extracted from the video pictures (automatic tracking) using a video-based motion analysis system (Peak Motus Motion Measurement System). The three-dimensional (3D) coordinates the eight reflective markers located on the back of the trunk were transformed from the Peak reference frame to local reference frames embedded in the middle and lower trunk (Figure 2). Considering the middle and lower trunk as adjacent segments of a joint, the anatomical joint (AJ) angles between the two segments is the relative orientation of the two segment-embedded local reference frames (see Additional file 1, [36]). The three components of the AJ angles represent the rotations about the medio-lateral axis (flexion/extension angle), antero-posterior axis (lateral flexion angle), and longitudinal axis (twisting angle). For each subject, the AJ angles obtained during serving trials were expressed as the angular deviation from the AJ angles recorded at standing posture (i.e., the AJ angles at standing posture are all 0°). The dependent variables for trunk motion were the maximal extension, left lateral flexion, and left and right twisting angles during a serve. For each subject, average normalized EMG values and maximum AJ angles over two trials of the same serve type were used for subsequent analysis.

### Data Analysis

For each serve type, phase and skill level combination, mean and standard deviation were computed for each variable of interest. For each muscle, the EMG parameters were compared using a 2 × 3 × 4 (Skill × Serve type × Phase) multivariate analysis of variance (MANOVA) with repeated measures on the last two factors. Separate univariate tests were performed for follow up testing when appropriate, and Bonferroni's procedure was used to adjust the overall type I error rate. To determine if significant variations existed among skill groups, serve types and trunk motions in the maximal AJ angle, a 2 × 3 × 4 (Skill × Serve type × Trunk motion) ANOVA with repeated measures on the last two factors was performed. An a-priori alpha level of 0.05 and an a-priori beta level of 0.20 were used in this study.

## Results

### Muscle Activation

**Figure 4:**
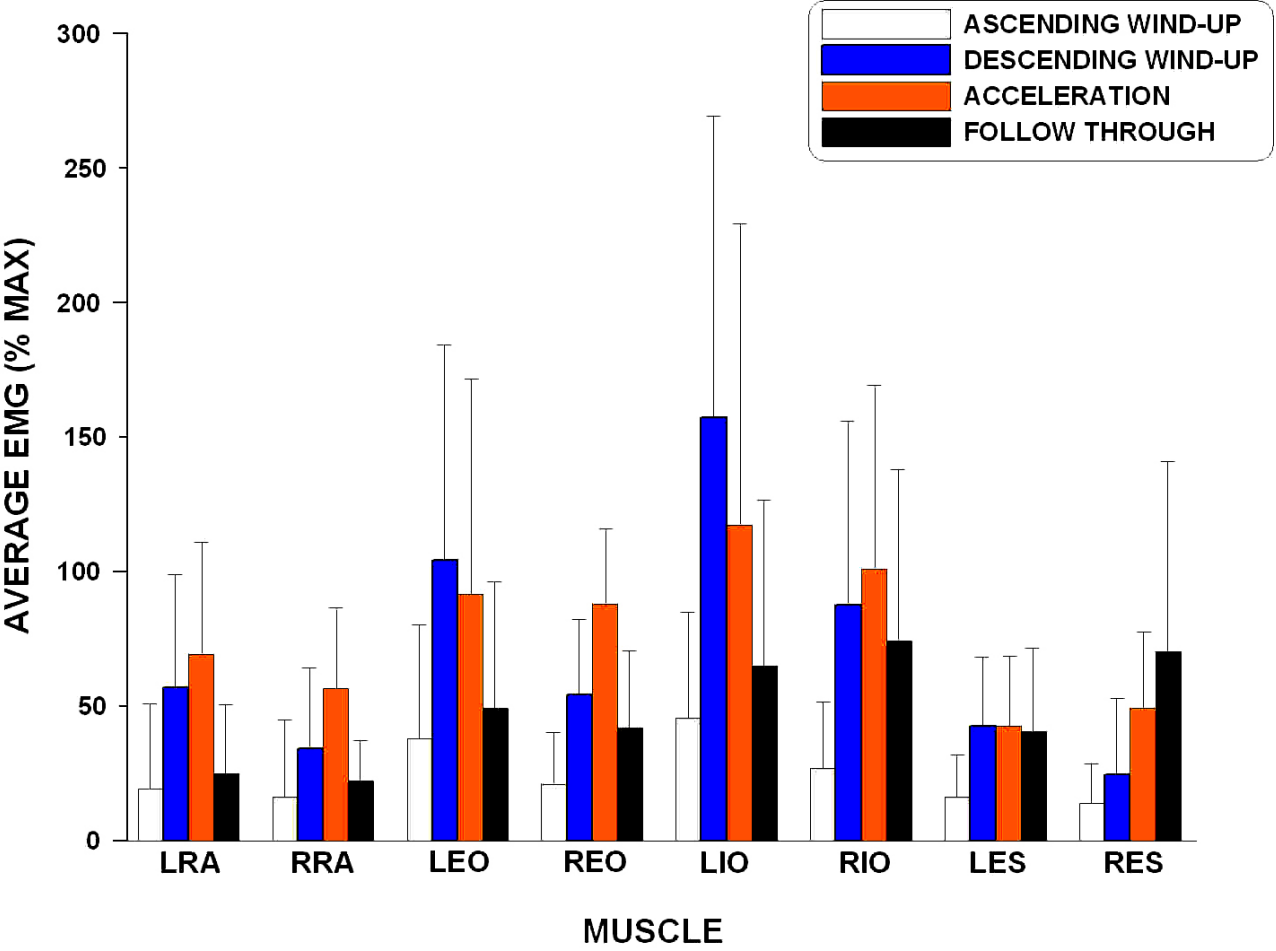
**Average EMG levels of different muscles during different phases**.

**Figure 5:**
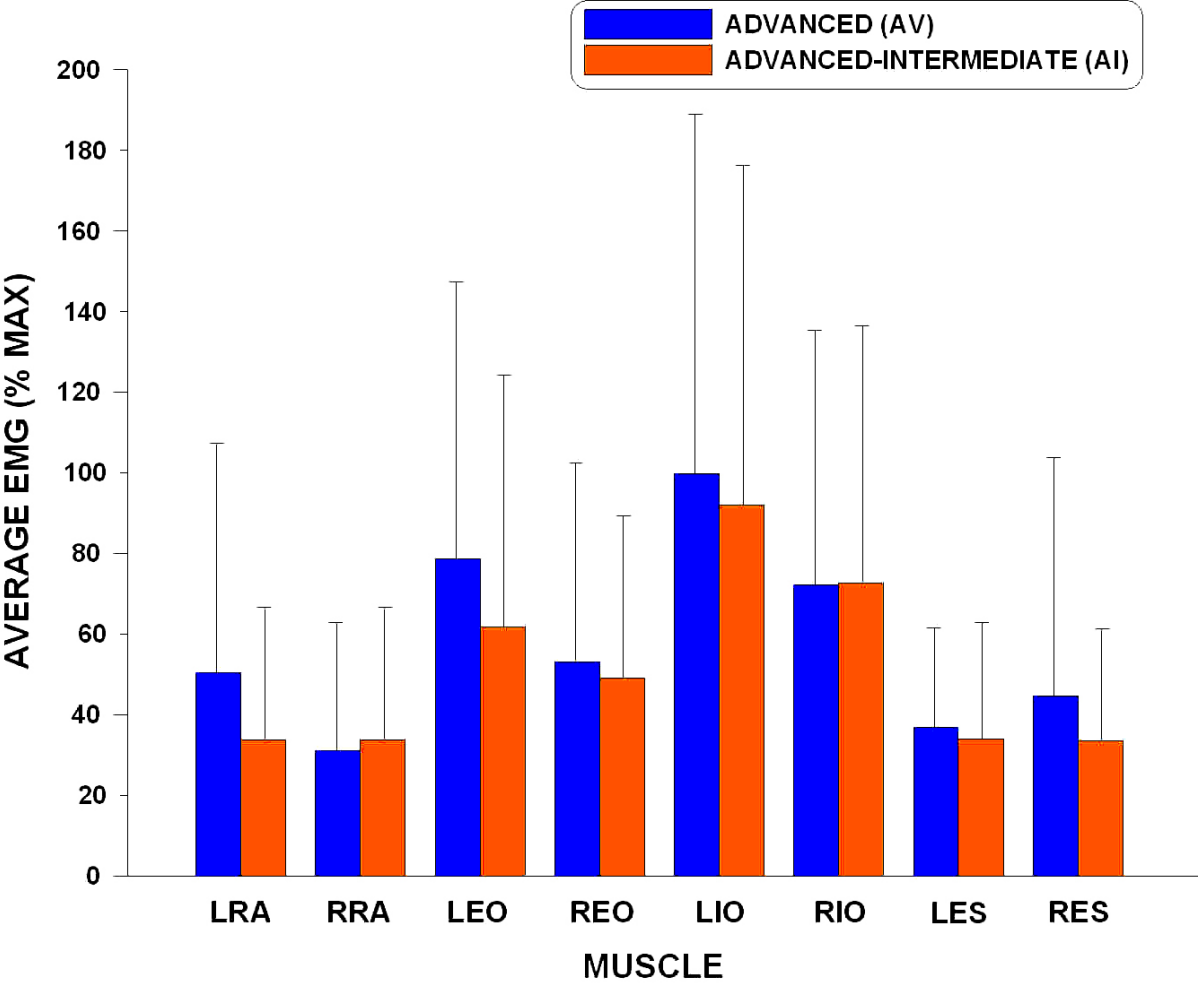
**Average EMG levels of different muscles for the two skill groups**.

As expected, a significant main effect for the phase was found in the average EMG level in each of the muscles monitored (p < 0.001). In addition, no significant main effect for the serve type or inter-factor interaction was found in any of the muscles (p > 0.695). In general, the lower trunk muscles become active toward the end of the ascending windup phase (Figure 4). For most muscles tested, the largest average EMG levels were observed in either the descending windup or acceleration phases. When comparing overall muscle activation during a tennis serve between the two skill groups, the subjects in the AV group generally exhibited greater muscle activation than the subjects in the AI group (Figure 5).

#### Rectus Abdominis

A significant main effect for the skill was found in the LRA activity (p = .008). The AV group showed significantly higher LRA activation than the AI group. Regardless of the serve type, the activation patterns of the LRA and RRA are quite similar - activations in the descending wind-up and acceleration phases are greater than the ascending windup and follow-through phases (Table 1).

**Table 1:** Average (SD) EMG (%MAX) during different phases for different serve types and muscles for subjects of different skill levels.

		**Flat**				**Topspin**				**Slice**			
**Muscle**	**Skill**	**AWU**	**DWU**	**ACC**	**FT**	**AWU**	**DWU**	**ACC**	**FT**	**AWU**	**DWU**	**ACC**	**FT**

Left RA*	AV	25 (45)	62 (34)	92 (89)	28 (28)	28 (44)	59 (40)	79 (71)	25 (24)	24 (41)	70 (59)	87 (90)	26 (16)
	
	AI	12 (12)	50 (39)	47 (22)	21 (21)	10 (7)	55 (48)	64 (35)	36 (44)	15 (13)	43 (33)	41 (28)	11 (6)

Right RA	AV	20 (38)	37 (43)	56 (30)	20 (11)	20 (35)	24 (16)	47 (34)	15 (7)	21 (40)	33 (28)	53 (34)	26 (23)
	
	AI	8 (4)	40 (35)	59 (34)	23 (14)	10 (6)	36 (32)	65 (50)	23 (14)	18 (26)	35 (26)	60 (43)	26 (19)

Left EO	AV	44 (50)	121 (86)	110 (80)	5 (40)	53 (61)	100 (68)	109 (78)	52 (35)	42 (49)	115 (100)	101 (57)	46 (24)
	
	AI	30 (30)	104 (95)	67 (56)	43 (35)	25 (23)	86 (60)	89 (65)	69 (95)	32 (29)	96 (84)	67 (50)	33 (29)

Right EO	AV	25 (26)	54 (32)	93 (70)	47 (36)	26 (31)	55 (44)	79 (61)	37 (27)	20 (21)	59 (36)	100 (80)	42 (23)
	
	AI	18 (10)	58 (16)	88 (60)	38 (21)	17 (10)	51 (14)	93 (61)	48 (35)	18 (7)	48 (14)	72 (53)	39 (34)

Left IO	AV	51 (41)	183 (144)	118 (61)	59 (38)	59 (61)	150 (94)	111 (61)	52 (36)	40 (27)	168 (127)	136 (91)	73 (68)
	
	AI	42 (43)	151 (113)	91 (49)	55 (26)	31 (24)	143 (97)	138 (84)	109 (123)	48 (35)	146 (119)	108 (64)	43 (9)

Right IO	AV	30 (28)	69 (38)	116 (76)	88 (92)	29 (29)	81 (60)	100 (63)	66 (60)	24 (21)	82 (58)	105 (63)	76 (76)
	
	AI	25 (25)	97 (74)	100 (63)	72 (50)	26 (27)	96 (87)	93 (64)	81 (51)	28 (25)	102 (101)	89 (55)	63 (54)

Left ES	AV	21 (20)	51 (28)	42 (27)	41 (19)	19 (20)	47 (29)	35 (20)	37 (17)	17 (18)	48 (28)	44 (25)	42 (23)
	
	AI	14 (14)	38 (24)	45 (24)	40 (31)	12 (11)	31 (23)	44 (20)	52 (63)	13 (11)	38 (25)	48 (22)	33 (19)

Right ES	AV	17 (21)	34 (34)	46 (31)	72 (65)	17 (22)	38 (37)	45 (34)	67 (66)	15 (17)	31 (39)	56 (55)	99 (142)
	
	AI	9 (4)	12 (5)	46 (28)	59 (21)	11 (6)	13 (8)	58 (30)	59 (22)	13 (9)	17 (10)	46 (16)	63 (25)

Abbreviations: AWU - ascending windup, DWU - descending windup, ACC - acceleration, FT - follow-through, RA - rectus abdominis, EO - external oblique, IO - internal oblique, ES - erector spinae, AV - advanced, AI - advanced-intermediate.

* Significant difference between AV and AI (p < 0.05).

#### External Oblique

Different patterns of activation were observed in the two EO muscles (Table 1). In general, the subjects in the AV group exhibited greater LEO activity than the subjects in the AI group and the difference was a statistical trend (p = 0.055).

#### Internal Oblique

Among the muscles examined, the IO muscles have the largest overall EMG levels (Figure 4). Different patterns of activation were observed in the two IO muscles (Table 1). The LIO was generally more active than the RIO throughout a serve except in the follow-through phase. Very high activation levels (average EMG levels greater than 100% max) were found in the descending windup and acceleration phases in the LIO.

#### Erector Spinae

Moderate activity was observed in all phases except the ascending windup for both ES muscles and the descending windup phase for the RES (Table 1). Different patterns of activation were observed in the two ES muscles (Figure 4). Instead of a relatively constant average EMG level after the ascending wind-up phase observed in the LES, the activity of RES increased steadily from ascending wind-up to follow-through phase. Although not statistically significant (p = 0.069), the AV group showed greater RES activation than the AI group (Table 1).

### Maximum AJ Angles

**Figure 6:**
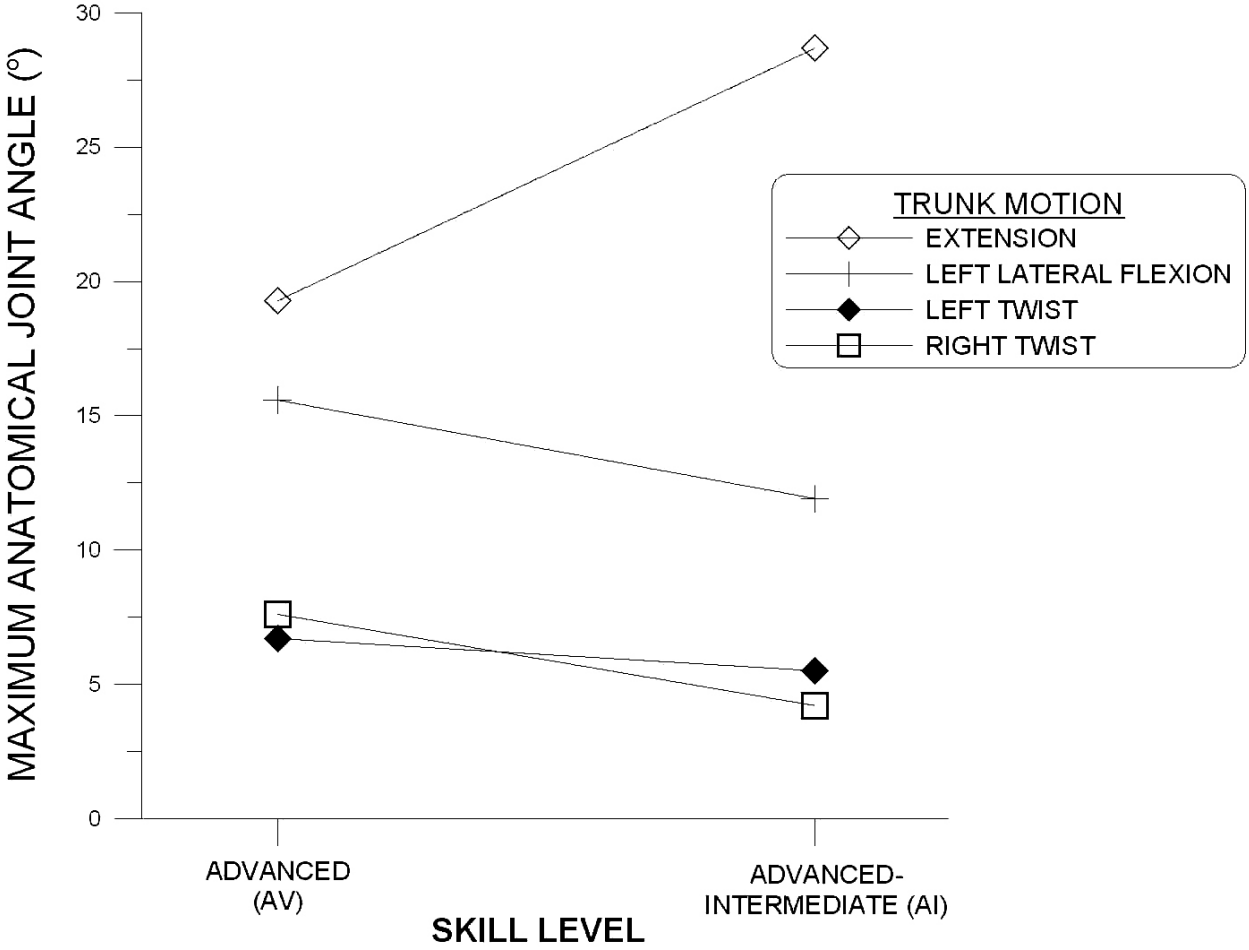
**Interactions between skill level and trunk motion in maximum anatomical joint angle**.

The repeated measures ANOVA performed on maximum AJ angles revealed a significant main effect for trunk motion (p < 0.001). This simply means that the middle-lower trunk ROM is not the same in different principle planes (Table 2). In addition, a significant interaction was found between trunk motion and skill factors (p < 0.001) (Figure 6). The AV group exhibited greater maximum AJ angles in all trunk motions measured except the extension. No significant main effect for serve type was detected.

**Table 2:** Mean (SD) maximum anatomical joint angles in degrees.

**Serve type**	**Flat**		**Top-spin**		**Slice**	
Motion*	**AV**	**AI**	**AV**	**AI**	**AV**	**AI**

Extension^#^	19.3 (11.9)	27.5 (10.9)	19.3 (10.6)	31.9 (17.2)	20.0 (10.1)	26.9 (7.6)

Left lateral Flexion^#^	16.0 (4.1)	12.3 (5.2)	15.5 (5.4)	10.9 (4.8)	15.4 (5.3)	12.4 (8.4)

Left Twisting	7.9 (4.3)	6.8 (3.8)	6.8 (4.1)	5.4 (0.96	5.5 (3.8)	4.3 (3.1)

Right Twisting	5.6 (4.6)	4.2 (1.8)	6.1 (6.9)	4.1 (0.6)	11.2 (12.7)	4.1 (3.1)

* Significant difference among different motions (p < 0.01).

^# ^Significant difference between AV (advanced) and AI (advanced intermediate) groups (p < 0.05).

The ANOVA also revealed significant differences between the two skill levels for the extension and left lateral flexion (Table 2). For the extension motion, the AV group exhibited a significantly smaller maximum AJ angle than the AI group (p = 0.018) while the opposite was observed in the left lateral flexion (p = 0.038).

## Discussion

When executing a tennis serve, vigorous movement of the trunk helps to generate as much angular momentum as possible and transfer it to the racquet [37]. Dynamic stability of the spine is essential to prevent low back dysfunction [38,39] and is associated with sufficient strength and endurance of the trunk stabilizing muscles and appropriate activation sequencing of the trunk muscles [40]. Spinal stability is also increased with either an increased coactivation of antagonistic counteracting trunk muscles or an increased intra-abdominal pressure during the static condition [38,41]. The primary focus of this study was to examine the role of lower trunk muscles in providing dynamic stability of the lumbar spine during a tennis serve and to speculate on the lumbar spine loads during a tennis serve using lower trunk muscle activation and kinematics data.

### Muscle Activation

The hypothesis that greater activation levels in lower trunk muscles would be found in topspin serves when compared to the other serve types was not supported by our results. In general, the activation patterns of different muscles during a tennis serve are comparable to those reported by Chow et al. [33]. However, Chow et al. [33] used players with skill levels similar to the subjects in the AV group of the present study. For the purpose of this discussion, an average EMG level of less than 10% max is considered low. Muscle EMG values of approximately 50% and 100% max are considered moderate and high, respectively.

#### Rectus Abdominis

The RA muscles are active during trunk flexion motions like curl-up, sit-up or leg raising exercises [42]. Due to its vertical (longitudinal) alignment, the RA has minimal contribution to the production of torque in the transverse plane [43]. High activation level of anterior supporting muscles of the lower trunk including RA may lead to unfavorable forces on the spine [44]. It has been suggested that an average normalized EMG value to MVIC (maximal voluntary isometric contraction) below 30% is not considered very stressful to the spine structures [45]. During the descending windup and acceleration phases of a tennis serve, the EMG level of both RA muscles increased to 40% or higher (Table 1). The AV group showed a higher level of activation during the acceleration phase than the AI group. With the center of gravity of the upper body located behind the lumbar spine, the RA activity (co-contraction) in a hyperextended posture during the descending windup and acceleration phases can drastically increase the loads on the lumbar spine and lead to harmful stress to the lumbar spine structures.

#### External Oblique

The EO muscle is one of the anterior supporting muscles of the lower trunk that is also active during trunk flexion exercises [42]. However, it has been well recognized that the contralateral EO muscle is one of the main movers for axial rotation of the trunk [46,47]. An appreciable antagonistic activity of the ipsilateral EO during axial rotation was also reported in the literature [48]. In the present study, the co-contraction of bilateral EO muscles helps to stabilize the lumbar spine during the tennis serve. This type of co-contraction helps to increase the compressive load and lead to the torsional stiffness of the lumbar spine segments [49,50]. High activation of LRA and LEO for the right-handers in the present study indicated that these muscles acted as prime movers of twisting to the right occurred during the descending windup and early acceleration phases.

#### Internal Oblique

Ipsilateral IO is the agonist to the contralateral EO for the axial rotation of the trunk [46]. Similar to the EO muscles, an appreciable co-contraction of contralateral IO is common during trunk twists. Interestingly, bilateral difference in muscle activation is more pronounced in IO than in the EO [51]. One explanation is that the function of EO is more complicated than just acting as a prime axial rotator of the lower trunk [48,52]. Like the EO muscles, bilateral co-contraction of IO observed during a tennis serve helps to provide a stabilizing force to the lumbar spine.

#### Erector Spinae

The lumbar ES lies lateral to the multifidus muscle and forms the prominent dorsolateral contour of the back muscles in the lumbar region. The lumbar ES consists of two muscles - the longisimus dorsi and iliocostalis. The lines of action of these two muscles are mostly vertical (or longitudinal) and a bilateral ES contraction can act as a posterior sagittal extensor. However, when contracting unilaterally, these muscles can act as lateral flexors of the lumbar vertebrae [53]. During axial rotation, the ipsilateral ES is more active than the contralateral ES [54]. It also has been suggested that, during axial rotation, back muscles maintain the spinal posture and stabilize the lumbar spine [22,43].

In the present study, we found different patterns of muscle activation during a tennis serve for the two ES muscles. The LES is quite active throughout a tennis serve except during the ascending windup phase while sequentially increased activity from ascending windup to follow-through phases was observed in the RES (Table 1). It is obvious that, for the right-handed subjects in this study, the LES assisted the lateral flexion to the left after the ascending windup phase. To stabilize the trunk during an unbalanced posture in the follow-through phase, the RES becomes highly active during the follow-through phase. The bilateral ES co-contraction is more pronounced in the AV group. This is probably related to the greater left lateral flexion found in the AV subjects (Figure 6). In addition to bilateral co-contractions, front/back co-contractions exist throughout a tennis serve and are especially high toward to end of a serve. This may suggest that the lumbar spine is subject to large compression loads during the follow-through phase.

### Lower Trunk Motion

In-vivo techniques have been employed to measure spine kinematics during various physical activities [55-57]. However, it is not feasible to use these techniques in the present study because of the large ROM associated with a tennis serve. Alternatively, we used markers placed on the lower back to estimate lumbar spinal motion during a tennis serve. The major limitation of our procedures is the skin movement relative to the spine during lower trunk motions. Despite this limitation, the marker locations allow for reasonable estimation of relative motion between the middle and lower trunk during a tennis serve.

#### Extension

During the extension motion, the vertebral bodies undergo posterior sagittal rotation and a small posterior translation. A downward movement of the inferior articular processes and the spinous process is also involved which limited by bony impaction between spinous processes [58]. This type of impaction is accentuated when the joint is subjected to the action of the back muscles [59]. The maximal extension AJ angles obtained in the present study fall within the extension ROM values reported in the literature [56,60]. It should be emphasized that it may not be adequate to compare the AJ angles in this study with the ROM values reported by other investigators because of the differences in measuring techniques.

The reason why the AI group had significantly greater maximum extension AJ angles than the AV group is difficult to understand. One explanation is that, instead of relying on lumbar hyperextension like the AI subjects did, the subjects in the AV group relied more on the hyperextension of the upper trunk (i.e., thoracic spine) to achieve the overall trunk hyperextension needed for an execution of a tennis serve.

#### Left Lateral Flexion

The lateral flexion of the lumbar spine involves a complex and variable combination of lateral bending and rotatory movements of the inter-body joints and diverse movements of the zygapophysial (facet) joints. When compared to lateral bending ROM values for lumbar spinal motion segments measured by X-rays [60,61], the maximal left lateral flexion AJ angles are close to the summed value of each motion segment in the lumbar spine. The significantly greater maximal left lateral flexion AJ angle exhibited by the AV group indicates that highly-skilled right-handed players can reach for a greater height during a tennis serve because of the greater left lateral flexion. The significantly greater lateral flexion AJ angle corresponds to the significantly greater LRA activity found in the AV group. This implies that highly-skilled players are subjected to greater asymmetric loads on their lumbar spines due to the greater lateral flexion.

#### Axial Rotation

Axial rotation of the lumbar spine involves twisting of the intervertebral discs and impaction of zygapophysial joints. During axial rotation of an intervertebral joint, all the fibers of the annulus fibrosus which are inclined to the direction of rotation will be strained. The other half will be relaxed. Based on the observation that an elongation of collagen beyond about 4% of its resting length can lead to injury of the fiber, it has been estimated that the maximum range of rotation of an intervertebral disc without injury is about 3° [62]. The twisting ROM for each lumbar spinal motion segment ranges from 0 - 2° [61]. The maximal twisting AJ angles found in the present study are slightly greater than the ROM values reported in the literature. The maximal twisting AJ angles are considered small compared to the amount of shoulder movement during a tennis serve. This clearly indicates that the axial rotation of the trunk during a tennis serve is mostly from the twisting of the upper trunk.

#### Co-contractions

Both bilateral and abdominal/back co-contractions among lower trunk muscles are unavoidable during trunk movements because these muscles function as units to maintain the balance between mobility and stability of the spinal column. As a result, the lumbar spine is subjected to a large amount of compressive and torsional stress during athletic movements due to the co-contraction. Although the importance of torsional stress in the etiology of disc degeneration and prolapse is inconclusive [63,64], the link between high compressive load and low back injury and pain is well documented [65,66]. The activation patterns of the lower trunk muscles clearly demonstrate a high degree of co-contraction during a tennis serve, especially in the descending windup and acceleration phases. In addition to the compressive load, the hyperextension and lateral flexion of the trunk during various phases of a tennis serve may cause shear loads on the lumbar spine. Consequently, stresses upon the various anatomical structures may result in spinal injury and back pain.

### Practical Implications

The risk of spinal injury can be high for tennis players of different skill levels. It is well known that physical activity increases the amount of bone mineral in the skeleton [67,68]. Granhed et al. [69] found that intensive weight lifting would increase the bone mineral content in the lumbar vertebrae to an extent that the spine can tolerate extraordinary loads. Unlike most competitive tennis players, recreational players usually do not spend much time on training or supervised strength and conditioning programs. As a result, their vertebrae are not as strong as the trained individuals and are more susceptible to injury when subjected to large lumbar spinal loads. Although highly-skilled competitive players are likely to have stronger vertebrae when compared to recreational players, they are also susceptible to spinal injury due to other factors. Because competitive players complete a large number of serves in practices and competitions, the accumulative stress on the lumbar spine can be detrimental.

One of the risk factors that has been overlooked by sports medicine practitioners is the possible link between the "time of occurrence" and back injury. Adams et al. [70] measured the range of lumbar flexion of human subjects in the early morning and in the afternoon and the bending properties of cadaveric lumbar segments before and after creep loadings that simulate a day's activity. They concluded that lumbar discs and ligaments are at greater risk of injury in the early morning compared with later in the day. Although the focus of their study was on lumbar flexion, it does have implications to lumbar spinal motion in general. It seems reasonable to advise patients with history of back disorders to avoid activity that will put the lumbar spine in extreme range of motion such as the tennis serve in the early morning. To gain more insight into this issue, inclusion of the "time of occurrence" in future epidemiological studies of acute low back injury is recommended.

The heavy involvement of lower trunk muscles in the tennis serve reinforces the importance of abdominal and lower back exercises in the strength and rehabilitation programs designed for tennis players. Because most lower trunk muscles undergo eccentric contractions during selected phases of the serve, it is recommended that eccentric training is included in the conditioning programs. The strengthening of the lower trunk muscles not only will enhance performance, but the tennis players will also benefit in preventing low back injury and pain.

### Recommendations for future studies

1. Future studies should examine if there are differences in activation patterns of the lower trunk muscles during the tennis serve among players of different skill levels including beginners.

2. In addition to lower trunk muscles, other back muscles such as multifidus and thoracic erector spinae muscles can be examined.

3. The use of EMG techniques to identify the muscle activation characteristics that are associated with low back pain [71] can be explored in tennis players.

4. Several epidemiological studies on low back disorders among young competitive tennis players have been conducted. However, such data are not available for non-competitive players of different ages. Future effort should examine the incidence of low back injuries in recreational players.

## Abbreviations

Muscles

RA: rectus abdominis (LRA: left RA; RRA: right RA); EO: external oblique (LEO: left EO; REO: right EO); IO: internal oblique (LIO: left IO; RIO: right IO); ES: erector spinae (LES: left ES; RES: right ES).

Others

AJ angle: anatomical joint angle; ANOVA: analysis of variance; MANOVA: multi-variate analysis of variance; EMG: electromyography or "electromyographic"; ROM: range of motion.

## Competing interests

The authors declare that they have no competing interests.

## Authors' contributions

JWC contributed to conception and design, acquisition of data, analysis and interpretation of data, and prepared the manuscript with the assistance of the other authors. Both SAP and MDT contributed to conception and design, acquisition of data, and revised the manuscript critically for important intellectual content.

## Supplementary Material

Additional file 1 **Appendix**. **Computation of Anatomical Joint Angles.** Click here for file
