# Supplementary material for: Lower trunk kinematics and muscle activity during different types of tennis serves
Source: Sports Med Arthrosc Rehabil Ther Technol. 2009 Oct 13;1:24. doi: 10.1186/1758-2555-1-24 (PMC2770553; doi:10.1186/1758-2555-1-24)
Supplement: Additional file 1 — Appendix. Computation of Anatomical Joint Angles. [file 1758-2555-1-24-S1.PDF]

APPENDIX Computation of Anatomical Joint Angles

Using the markers attached to the back of the subject (see Figure 2), a local coordinate system was defined for each trunk segment under consideration. Referring to the figure below, the unit vectors defining the orientations of the middle trunk (subscript M) and lower trunk (subscript L) segments are as follows:

$\mathbf{i}_M$ = a unit vector directed from Marker 2 to Marker 1
$\mathbf{k}_M$ = a unit vector directed from Marker 4 to Marker 3
$\mathbf{j}_M = \mathbf{k}_M \times \mathbf{i}_M$ (cross product)
$\mathbf{i}_L$ = a unit vector directed from Marker 6 to Marker 5
$\mathbf{k}_L$ = a unit vector directed from Marker 8 to Marker 7
$\mathbf{j}_L = \mathbf{k}_L \times \mathbf{i}_L$ (cross product)

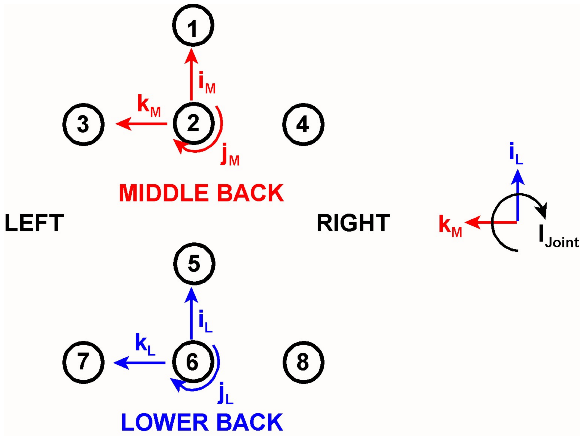

For computation purposes, the middle and lower trunk are considered as the proximal and distal segments, respectively. The following equations were used to compute relative orientation of middle trunk and lower trunk (anatomical joint angles):

$$\mathbf{I}_{Joint} = (\mathbf{k}_M \times \mathbf{i}_L)/|\mathbf{k}_M \times \mathbf{i}_L| \qquad [1]$$

$$\alpha = \sin^{-1}[\mathbf{I}_{Joint} \cdot \mathbf{i}_M] \qquad [2]$$

$$\beta = -\sin^{-1}[\mathbf{k}_M \cdot \mathbf{i}_L] \qquad [3]$$

$$\gamma = \sin^{-1}[\mathbf{I}_{Joint} \cdot \mathbf{k}_L] \qquad [4]$$

where α, β, and γ represent flexion/extension, left/right lateral flexion, and left/right twisting angles, respectively. Dot product was used in Eq. 2-4 to determine the angle between the 2 vectors involved.
